# Expression of Three *Related to ABI3/VP1* Genes in *Medicago truncatula* Caused Increased Stress Resistance and Branch Increase in *Arabidopsis thaliana*

**DOI:** 10.3389/fpls.2020.00611

**Published:** 2020-05-25

**Authors:** Shumin Wang, Tao Guo, Zhen Wang, Junmei Kang, Qingchuan Yang, Yixin Shen, Ruicai Long

**Affiliations:** ^1^College of Agro-Grassland Sciences, Nanjing Agricultural University, Nanjing, China; ^2^Institute of Animal Science, Chinese Academy of Agricultural Sciences, Beijing, China; ^3^College of Grassland Science, Beijing Forestry University, Beijing, China

**Keywords:** RAV, *Medicago truncatula*, abiotic stress response, ABA, cold tolerance, branch number increasement

## Abstract

Related to ABSCISIC ACID INSENSITIVE3 (ABI3)/VIVIPAROUS1(VP1)(RAV) transcription factors, which encode a B3 domain and an APETALA2(AP2) domain, belong to the APETALA2/ethylene-responsive element binding factor(AP2/ERF) or B3 superfamily and play an important role in regulating plant growth and development and responding to abiotic stress. Although there have been many functional studies on RAV, the functional differences between RAVs are not clear. Therefore, in this study, the functional differences of RAVs of *Medicago truncatula* were analyzed. Based on sequence data from the plant transcription factor database and the *M. truncatula* genome database, we cloned three *RAV* genes from *M. truncatula*, named *MtRAV1*, *MtRAV2*, and *MtRAV3*. The cis-acting elements of these genes promoters were predicted, and the expression patterns of *MtRAVs* under exogenous conditions (4°C, NaCl, Polyethylene Glycol, Abscisic acid) were analyzed. *MtRAVs* transgenic *Arabidopsis thaliana* were obtained and subjected to adversity treatment. Subcellular localization results indicated that MtRAVs were located in the nucleus. A much lower expression level was observed for *MtRAV3* than the levels of *MtRAV1* and *MtRAV2* in *M. truncatula* for growth in normal conditions, but under 4°C or PEG and NaCl treatment, the expression level of *MtRAV3* was significantly increased. Only the *MtRAV3* overexpression transgenic plants showed strong cold resistance, but the overexpressed *MtRAV1* and *MtRAV2* transgenic plants showed no difference from wild type plants. *MtRAV* transgenic plants exhibited similar response to exogenous mannitol, NaCl, and ABA, and the expression of some adverse-related marker genes were up-regulated, such as *COLD REGULATED 414 THYLAKOID MEMBRANE 1 (COR414-TM1)*, *Arabidopsis thaliana drought-induced 21 (AtDI21)*, and *Arabidopsis thaliana phosphatidylinositol-specific phospholipase C (ATPLC)*. *MtRAVs* transgenic *Arabidopsis thaliana* exhibited increasing of branch number. These results indicated that there was some function redundancy during MtRAVs proteins of *M. truncatula*, and MtRAV3 has increased function compared to the other two genes. The results of this study should provide the foundation for future application of *MtRAVs* in legumes.

## Introduction

RAV (Related to ABI3/VP1) transcription factors belong to the AP2 or B3 superfamilies. These proteins encompass an AP2 domain in the N-terminal region, and a B3 domain in the C-terminal region, having two unrelated DNA binding domains, a property that is unique among transcription factors (Swaminathan et al., 2008; Mizoi et al., 2012). RAVs and some TFs, such as GRAS and NAC, are plant-specific transcription factors, and much less is known about these plant-specific proteins compared to TF families that have orthologs in animals and bacteria (Riechmann et al., 2000). AP2/ERF proteins are classified into four subfamilies: AP2, DREB (for dehydration response element-binding protein), ERF, and RAV (Mizoi et al., 2012). The B3 superfamily also encompasses four distinct protein families, including ARF (auxin response factor), LAV (leafy cotyledon2 [LEC2]–abscisic acid insensitive three [ABI3]–val), REM (reproductive meristem) and RAV families (Swaminathan et al., 2008). AP2 and B3 proteins were shown to be involved in the regulation of biotic and abiotic stress responses, plant growth and development (Park et al., 2001; Zhang et al., 2005; Swaminathan et al., 2008). Transcription factors, such as AP2, DREB (CBF), ERF and LAV (ABI) families in plants, regulate the expression of defense genes in responses to biotic and abiotic stresses, seed maturation and seedling growth (Swaminathan et al., 2008; Dietz et al., 2010; Mizoi et al., 2012).

Compared with other subfamilies of the AP2/ERF superfamily, the plant RAV family is a relatively small transcription factor family, with orthologs found only in higher plant species. There are seven members in *Arabidopsis thaliana*, five in *Glycine max*, and three in *M. truncatula*. Some genes of the RAV family have been studied in plants such as *Arabidopsis thaliana*, *Zea mays*, *Oryza sativa*, *Solanum lycopersicum*, and *Capsicum annuum* (Hu et al., 2004; Lee et al., 2010; Li et al., 2011; Min et al., 2014; Zhu et al., 2018), with functions related to growth and development, hormone regulation, and response to adverse situation (Endres et al., 2010; Matías-Hernández et al., 2014).

Previous research revealed that the expression levels of *GhRAV1*, *ZmRAV1* and *GmRAV-03* genes were up-regulated by salt, polyethylene glycol (PEG), or dehydration, respectively, in *Gossypium hirsutum*, *Zea mays*, and *Glycine max* (Min et al., 2014; Li et al., 2015; Zhao et al., 2017). *AtRAV1* or *AtRAV2*, *GmRAV-03*, *ZmRAV1*, *CaRAV1*, and *SlRAV2* transgenic plants exhibited increased resistance to high salt and drought compared to wild-type, by activation of resistance-related genes in the pathway of response to abiotic stress (Sohn et al., 2006; Li et al., 2011; Min et al., 2014; Mittal et al., 2014; Zhao et al., 2017).

Cold stress is an important factor that affects plant growth and distribution. *RAV* play a regulatory role in the *CBF*-*RAV*-*ERF*-*PR* signaling cascade, part of the defense mechanism related to biotic stress response (Li et al., 2011). RAV1 functions as a cold-responsive transcription factor that may also be involved in a regulatory pathway independent of DREB/CBF (Fowler and Thomashow, 2002). The *BnaRAV-1-HY15* gene was induced by cold treatment (Zhuang et al., 2011), and a separate report determined that cold stress induced nearly half of the RAV subfamily transcripts (Du et al., 2016). The overexpression of *SlRAV2* in *Solanum lycopersicum* resulted in stronger tolerance to cold stress (Li et al., 2011). When *Zea mays* seedlings were immersed in Hoagland solution supplemented with 25 mM LiCl, the expression of *ZmRAV1* was induced initially and remained at high level for 24 h (Min et al., 2014). These results suggested that RAV proteins play important roles in the salt, drought and cold response.

RAV has been reported to be involved in ABA signal pathways. For example, *RAV* was induced rapidly by ABA in some plants such as *Glycine max*, *Zea mays*, *Gossypium hirsutum*, and *Capsicum annuum* (Sohn et al., 2006; Min et al., 2014; Li et al., 2015; Zhao et al., 2017). However, *GmRAV-03* transgenic plants were insensitive to exogenous ABA treatment, although ABA dramatically induced the expression of all *GmRAV* genes (Zhao et al., 2017). RAV also was shown to be involved in signaling crosstalk of other plant hormones, such as GA, BR, and ethylene (Zhu et al., 2018).

*AtRAV1* and its orthologs in other species have been reported to participate in various biological processes such as plant growth and development. In *Arabidopsis thaliana*, overexpression of *TEM* (belong to RAV subfamily) genes resulted in retarded-growth phenotypes of transgenic plants, through repressing the expression of GA4 biosynthetic genes (Osnato et al., 2012). *Arabidopsis thaliana AtRAV1* was reported to act as a positive regulator to cause leaf senescence (Woo et al., 2010). Transgenic tobacco overexpressing *GmRAV* exhibited slower plant growth rate, delayed flowering time, decreased root elongation, down-regulated photosynthetic rate, and decreased chlorophyll contents of leaves (Zhao et al., 2008; Lu et al., 2014). In addition, both *ZmRAVL1* RNAi and knockout lines exhibited smaller leaf angle in lower, middle, and upper leaves compared with wild-type plants (Tian et al., 2019). These results indicate the involvement of RAV proteins in the complex regulation network of plant growth and development.

However, there is a certain difference or even the opposite function during RAV TFs of different species or the same species. *AtRAV1*-overexpressing transgenic *Arabidopsis thaliana* displayed strong growth inhibition accompanied by early senescence, while the *AtRAV1L* or *AtRAV2*-overexpressing transgenic plants did not show any alterations in the growth and development compared to wild type (Fu et al., 2014). *Gossypium hirsutum GhRAV1* transgenic *Arabidopsis thaliana* displayed weaker resistance than wild type to the treatment of NaCl or drought stress (Li et al., 2015). While *Zea mays ZmRAV1* transgenic *Arabidopsis thaliana* enhanced salt and osmotic stress tolerance compared to the wild type, through increasing survival rate, longer primary roots and lower relative electrolyte leakages (Min et al., 2014).

*RAVs* are important genes affecting plant growth and development, hormone regulation, and response to adverse situation. Although there have been many reports on the functions of RAV family proteins, new functions have been discovered continuously in recent years, suggesting a strong exploration potential. There have been few systematic studies on the relationships between RAVs, and it is not clear to what extent there is functional redundancy between genes. Since *M. truncatula* is a model plant, its genome information is relatively complete, so used *M. truncatula* to conduct a systematic study on the RAV family. The purpose of this study was: (I) to investigate the functions of RAV genes in *M. truncatula* and (II) to further explore the regulatory network involved in *RAV* gene effects on stress tolerance, particularly by analysis of the expression characteristics of downstream genes. The findings of this study on *M. truncatula RAVs* can be extended to other legumes such as alfalfa, laying a foundation for research into the basic functions of RAVs for improved utilization of genetic resources.

## Materials and Methods

### Identification and Isolation of *M. truncatula RAVs*

Transcription factor prediction of PlantTFDB and the *M. truncatula* genome information provided by NCBI indicated that there are three *RAV* genes in *M. truncatula*, *MtRAV1*, *MtRAV2* and *MtRAV3*. None of the genes contain introns. *M. truncatula* genomic DNA was used as the template, and MtRAV1P-F/R, MtRAV2P-F/R, and MtRAV3P-F/R (Supplementary Table S1) were used as the primers to amplify the CDS regions of *MtRAV1*, *MtRAV2*, and *MtRAV3.*

### Bioinformatics Analysis

The presence of cis-acting elements in the promoters of the three *MtRAV* genes was analyzed in Plant CARE. We performed a multiple alignment analysis with ClustalX (2.0) and constructed a phylogenetic tree with the neighbor-joining (NJ) method. The phylogenetic tree diagram was constructed using MEGA7.0 Bootstrap.

### Subcellular Localization Analysis in *M. truncatula*

The obtained sequences of the CDS regions of *MtRAV1*, *MtRAV2*, and *MtRAV3* were PCR-amplified with SAT-RAV-F and SAT-RAV-R primers (Supplementary Table S1). The resulting DNA fragments were fused to plasmid pSAT6-GFP cut with NCO I in the presence of seamless ligase presence to create 35S:*MtRAVs*-GFP fusions driven by the CaMV35S promoter. After confirmation of the fusion plasmids by DNA sequencing, the recombinant plasmids were transformed into protoplasts of *M. truncatula* mesophyll cells, with the empty pSAT6-GFP vector as a control. Mesophyll cell protoplasts were transformed as previously described (Yoo et al., 2007). After 18 h of darkness, the transformed protoplasts were monitored by confocal laser scanning microscope.

### Plant Materials and Growth Conditions

The wild type used in this study was *Arabidopsis thaliana* Columbia (Col-0). Seeds were surface-sterilized with 10% NaClO aqueous solution for 10 min, followed by washing eight times with sterile water. Seeds were germinated on 1/2 MS Phytagar plates (pH = 5.8) supplemented with the appropriate antibiotics, vernalized for 2-day in the dark at 4°C, then plates were transferred to the light at 22°C and incubated under a 16 h light/8 h dark cycle. Upon emergence of the third and fourth leaves of seedlings, seedlings were transferred to sterilized soil to continue to grow or to 1/2 MS Phytagar plates containing other treatment materials.

*M. truncatula* R108 seeds were provided by our lab. After breaking dormancy, seeds were surface-sterilized with 75% alcohol, and then washed eight times with sterile water. These sterilized seeds were placed on aseptic filter paper to sprout, and then were cultivated in Hoagland nutrient solution for 21 days or transferred to sterilized soil for continued growth under conditions of 16 h light at 26°C/8 h dark at 24°C.

### Treatment of Various Abiotic Stress and ABA

*M. truncatula* R108 seedlings grown in Hoagland nutrient solution for 21 days were sprayed with ABA and then sampled at 0, 2, 4, 8, 12, and 24 h. For treatment with salt and PEG abiotic elicitors, *M. truncatula* 21-day-old seedlings were transferred to Hoagland nutrient solution containing 200 mM NaCl or 15% PEG and incubated for 24 h. For cold treatment, 21-day-old *M. truncatula* seedlings grown in soil were placed in a low temperature illumination incubator at 4°C for 24 h.

### Real-Time Quantitative PCR Analysis

Plant total RNA was isolated with the EastepTM Super Total RNA Extraction Kit and then cDNA synthesis was performed using the PrimeScript RT reagent Kit with gDNA Eraser (Perfect Real Time). Total RNA and cDNA were assayed by electrophoresis and quantified by NanoPhotometer 2000. The cDNA was mixed with TB Green Premix Ex Taq for qRT-PCR using an Applied Biosystems (ABI) 7300 Real Time PCR System. The obtained values of the corresponding gene expression were normalized according to the amounts of *Mtactin* or *Atactin* for the comparative ΔΔCt method. The primers used for quantitative realtime PCR are presented in Supplementary Table S1. All quantitative analysis were performed on three independent biological replicates.

### Generation of Transgenic Plants

The obtained complete sequences of the CDS regions of *MtRAV1*, *MtRAV2* and *MtRAV3* were cloned into the T5 pEASY^®^-T5 Zero Cloning vector. Subsequently, these target gene fragments were amplified with the primers listed in Supplementary Table S1, and then were ligated into the *Nco*I-digested pCAMBIA3301 (p3301) vector that harbors the CaMV35S promoter in the presence of seamless ligase. To generate the *MtRAVs*-overexpressing transgenic plants, the constructs were transformed into *Agrobacterium tumefaciens* (GV3101), and introduced by plant transformation using the floral dipping method (Clough and Bent, 1998). All T1 generation transgenic *Arabidopsis thaliana* lines of three *MtRAV* genes were sampled and examined the mRNA transcription level by qRT-PCR methond (Supplementary Figure S4). All experiments were performed with homozygous lines in the T3 generation.

### Tolerance Assays Under Stress Conditions

For the ABA, NaCl, and osmotic stress tolerance assays, the seedlings grown on 1/2 MS medium in the normal condition of 16 h light/8 h dark cycle for 5 days and then transferred to 1/2 MS medium containing 20 μm ABA, 200 mM NaCl, or 500 mM mannitol. Three transgenic lines of each *MtRAV* gene were used for various treatment, include 35S:*MtRAV1-*#9, #11 and #4, 35S:*MtRAV2-*#7, #2 and #5, 35S:*MtRAV3-*#12, #7 and #5 lines (Supplementary Figure S4). All assays were performed in triplicate.

For the NaCl stress assay, the results were observed after seedlings grew for 3 days on the 1/2 MS medium containing 200 mM NaCl, and the number of surviving seedlings was counted. For the ABA and osmotic stress assays, 12 and 7 days after treatment, root length and fresh weight were measured, respectively.

For the cold tolerance assay, wild-type and transgenic plants were grown on 1/2 MS medium containing 1% agar and grown at 22°C for an additional 18-day. The plates were transferred to a -15°C growth chamber and incubated for 2 h, then were incubated for 24 h at 22°C before counting the number of surviving seedlings. Three transgenic lines of each *MtRAV* gene were used for various treatment, include 35S:*MtRAV1-*#9, #11 and #4, 35S:*MtRAV2-*#7, #2 and #5, 35S:*MtRAV3-*#12, #7 and #5 lines. All assays were performed in triplicate.

## Results

### Identification of *RAV* Genes From *M. truncatula*

**FIGURE 1:**
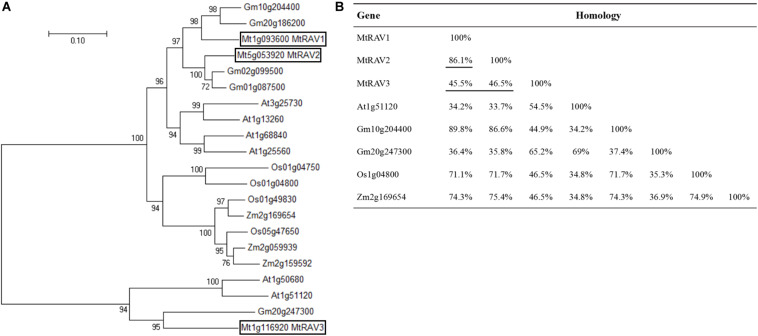
Phylogenetic tree analysis and homologous relation of RAV proteins from different species. **(A)** Phylogenetic tree analysis of 21 RAV proteins. Sequences were obtained from NCBI. Tree was constructed using neighbor-joining method in MEGA7.0 software. **(B)** Sequence homology of RAV proteins in the indicated species.

To identify RAV transcription factors in *M. truncatula*, we performed BLAST searching in the NCBI database by using the publicly known RAV subfamily TF protein sequence AT1G13260 as a query sequence. We identified three homologous genes, MTR_1g093600, MTR_5g053920, and MTR_1g116920, now named as *MtRAV1, MtRAV2*, and *MtRAV3*, respectively. These genes contain both AP2 and B3 domains. Phylogenetic tree analysis was performed on these three genes plus 17 previously described RAV genes using neighbor-joining method in MEGA7.0 software (Figure 1A). *M. truncatula* is a model plant of leguminosae, and the RAV proteins sequences are close to those of other leguminosae (Figure 1B). *Glycine max* RAV (Gm10g204400) has higher homology with MtRAV1 and MtRAV2, with alignment of 89.8 and 86.6% (Figure 1B), respectively. MtRAV3 and GmRAV (Gm20g247300) share 65.2% homology (Figure 1B). There is 86.1% homology between MtRAV1 and MtRAV2, while MtRAV3 has only 45.5 and 46.5% homology with MtRAV1 and MtRAV2 (Figure 1B), respectively. Thus, there may be functional differences between the three *MtRAV* genes.

### Expression Analysis of *RAV* Genes in *M. truncatula*

**FIGURE 2:**
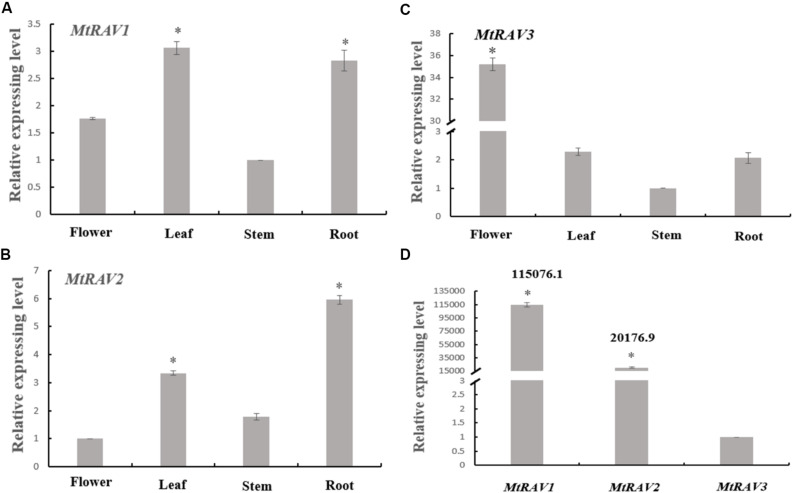
Expression of *MtRAVs* genes in *M. truncatula*. **(A–C)** Relative expression level analysis by qRT-PCR of *MtRAV* genes in different plant organs of *M. truncatula* grown for 52 days under conditions of 16 h light at 26°C/8 h dark at 24°C, when the flowers bloom. Flower, leaf, stem, and root samples were analyzed. **(A,C)** Expression analysis relative to the expression amounts of the *MtRAV1* and *MtRAV3* gene in the stem sample. **(B)** Expression analysis relative to the expression amounts of *MtRAV2* in flower. **(D)** The expression level difference analysis of the three *MtRAV* genes in the whole *M. truncatula* plant grown for 21 days by qRT-PCR. The results are relative to the expression amounts of *MtRAV3*. The data are shown as mean values ± SD (*n* = 3). Independent *t*-tests demonstrated that there was significant difference (^∗^*P* < 0.05).

Based on homology alignment result of the three *MtRAV* genes, we next examined the expression patterns of the three genes by qRT-PCR in the flower, leaf, stem, and root of *M. truncatula*. *M. truncatula* plants grown under conditions of 16 h light at 26°C/8 h dark at 24°C for 52 days were used and RNA samples were extracted from flowers, leaves, stems, and roots. These samples were then used in qRT-PCR analysis. All quantitative analysis were performed on three independent biological replicates. A histogram was constructed to present the expression patterns based on qRT-PCR data (Figure 2). The results showed that MtRAV proteins can be detected in all tissues of *M. truncatula* during the flowering development period, with different expression levels of the same gene in different tissues. For example, *MtRAV1* was expressed at a higher level in leaf, *MtRAV2* expression level was higher in root, and *MtRAV3* expression level was higher in flower samples. These results suggest that the three *MtRAV* genes may play different regulatory roles in *M. truncatula* plants. Next, we examined the relative expression level of the three *MtRAV* genes in the whole *M. truncatula* plant by qRT-PCR. RNA of 21-day-old *M. truncatula* seedlings were extracted and used in qRT-PCR analysis. The results revealed a wide range of expression levels of the three genes. The *MtRAV1* transcripts were highest in all tissues, compared to the amounts of *MtRAV2* and *MTRAV3* transcripts. The expression level of *MtRAV1* was 115,076-fold than that of *MtRAV3.* These results indicated that *MtRAV3* may play a particular role in the RAV subfamily in *M. truncatula*.

### Subcellular Localization of *MtRAVs* Genes

**FIGURE 3:**
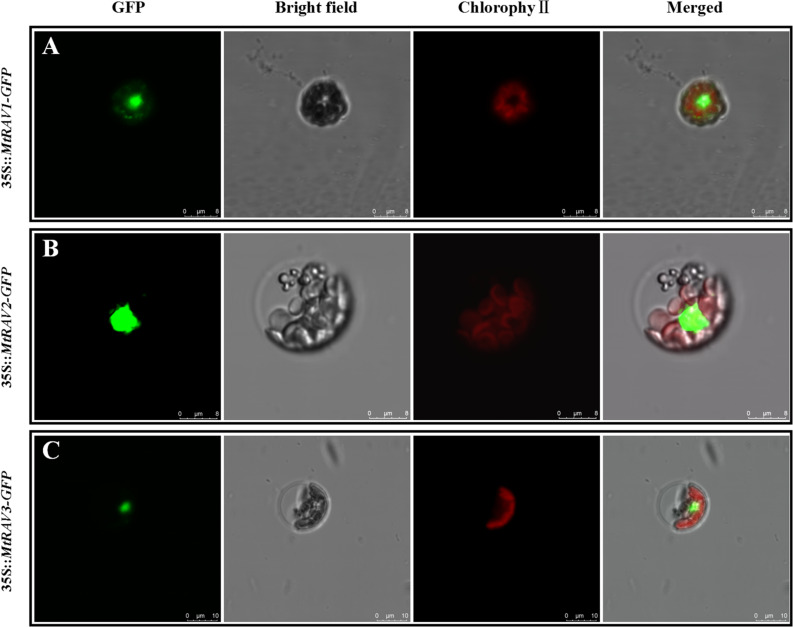
Subcellular localization of the MtRAV proteins. **(A,B)** The 35S:MtRAV-GFP fusion proteins, were delivered into protoplasmic cells of *M. truncatula* and transiently expressed, then visualized 18 h after transformation with confocal laser scanning microscopy. Under the green fluorescence excitation channel, MtRAV1-GFP and MtRAV2-GFP obviously localized to the nucleus (green). Scale bars = 8 μm. **(C)** MtRAV3-GFP also localized to the nucleus (green). Scale bars = 10 μm.

Sequence analysis using NCBI showed that *MtRAV* genes are transcription factors, so to confirm whether MtRAVs localize to the nucleus, we constructed 35S:*MtRAVs-GFP* fusion proteins. These were delivered into mesophyll protoplast cells of *M. truncatula* as described in a previous method (Yoo et al., 2007). Next, subcellular localization of GFP expression in *M. truncatula* protoplasts was observed by confocal microscopy 18 h after transformation of the fused plasmid pSAT6:*MtRAV*-*GFP*. The empty 35S:GFP vector was transformed as the control. The results revealed that *M. truncatula* RAV fusion proteins all localized in the cell nucleus (Figure 3), which suggests that these are nuclear proteins, and may act as transcription factors.

### Promoter Prediction and Analysis for *MtRAVs*

Since localization analysis revealed that all three RAV proteins are located in the nucleus, the promoter sequences were analyzed to predict potential cis-element sequences. We obtained the sequences of the promoters of the three *MtRAV* genes from NCBI, and then used PlantCARE to identify the cis-elements for the three *MtRAV* genes. Different cis-elements were identified in the *MtRAV* gene promoters (Table 1): Only *MtRAV1* contained three ABREs, which were absent in the *MtRAV2* and *MtRAV3* promoter sequences. However, MYC and BOX 4 elements were found in all three *MtRAV* gene promoters. Additionally, *MtRAV2* and *MtRAV3* contained MYB repeats, and TC-rich repeats and CGTCA-motif elements were found in *MtRAV1* and *MtRAV3* promoters. Analyses of these cis-elements in the promoters of the *M. truncatula RAV* genes provide insight into predicted functions.

**TABLE 1:** Distribution of abiotic stress-related cis-acting elements in *M. truncatula RAV* gene promoters.

Gene	ABRE	MYC	MYB	BOX4	W-BOX	TC-rich	CGTCA-
						repeats	motif
*MtRAV1*	3	2	0	1	1	1	1
*MtRAV2*	0	2	5	1	1	0	0
*MtRAV3*	0	1	3	1	1	1	1

*ABRE cis-acting elements can be bound by ABA, AREB, and ABF proteins; MYC elements can be bound by ICE proteins; MYB elements can be bound by MYB proteins; BOX4 is a light-responsive element; W-BOX can be bound by WRKY proteins; TC-rich repeats are involved in defense to biotic and abiotic stress; and CGTCA-motifs are related to MeJA-responsiveness.*

### Expression Analysis of *MtRAVs* to Abiotic Stress and ABA

**FIGURE 4:**
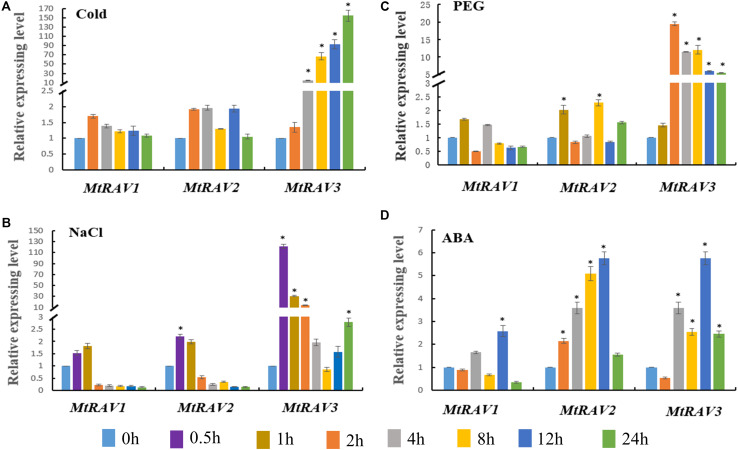
Expression profiles of *MtRAV* genes in plants treated with Cold, NaCl, PEG, and exogenous application of ABA. *MtRAV* mRNA expression levels were measured by qRT-PCR analysis. **(A)** For cold treatment, 21-day-old *M. truncatula* seedlings grown in soil were placed in a low temperature, illumination incubator at 4°C, and samples were taken at 0, 2, 4, 8, 12, and 24 h. **(B)** For NaCl treatment, 21-day-old *M. truncatula* seedling roots were immersed in Hoagland solution supplemented with 200 mM NaCl and samples were removed at 0, 0.5, 1, 2, 4, 8, 12, and 24 h. **(C)** For osmotic treatment, 21-day-old *M. truncatula* seedlings were transferred to Hoagland nutrient solution containing 15% PEG and samples were removed at 0, 1, 2, 4, 8, 12, and 24 h. **(D)** For ABA treatment, 100 μM ABA was sprayed on 21-day-old *M. truncatula* seedlings grown in Hoagland nutrient solution. Seedlings were sampled at 0, 2, 4, 8, 12, and 24 h. All experiments were replicated three times. Data are shown as the mean values ± SD. Independent *t*-tests demonstrated that there was significant difference (^∗^*P* < 0.05).

Based on the cis-element sequence analysis of the *MtRAV* genes promoter, we predicted that RAV proteins might be involved in abiotic stress and ABA signaling regulation pathways. Therefore, we further examined the expression profiles of *MtRAV* genes by qRT-PCR under ABA and abiotic stress treatment, including cold, NaCl, and PEG. All quantitative analysis were performed on three independent biological replicates. The results showed significant induction of *MtRAV3* by cold, salt, and PEG to higher levels than *MtRAV1* and *MtRAV2* (Figure 4). The expression amounts of *MtRAV3* were initially upregulated by low temperature 4°C and by 24 h, increased to 154-fold higher than the control plants grown under normal conditions (Figure 4A). As shown in Figure 4B, in response to exogenous treatment with 200 mM NaCl, the amounts of the three *MtRAV* transcripts were rapidly upregulated within an hour, and subsequently showed a gradual decline. The results indicated a rapid response of *MtRAVs* to salt stress. However, under the 15%PEG treatment, the up-regulation degree of *MtRAV3* was greater than those of *MtRAV1* and *MtRAV2* (Figure 4C). For exogenous 100 μM ABA treatment, the expression levels of *MtRAV1*, *MtRAV2*, and *MtRAV3* were induced, and by 12 h, reached levels that were more than twofold higher than the control plants without ABA treatment. These results suggest that *MtRAV3* was more sensitive to abiotic stress than *MtRAV1* and *MtRAV2*.

### Overexpression of *MtRAV3* Improved *Arabidopsis thaliana* Tolerance to Cold

The *MtRAVs* expression level of all T1-generation transgenic plants were detected by qRT-PCR, and three transgenic strains with the higher *MtRAVs* expression level were selected for subsequent assays of stress tolerance and phenotypic observation. The results showed that the higher *MtRAVs* expression level were respectively in the MtRAV1-#4/9/11 lines, MtRAV2-#2/5/7 lines and MtRAV3-#5/7/12 lines.

**FIGURE 5:**
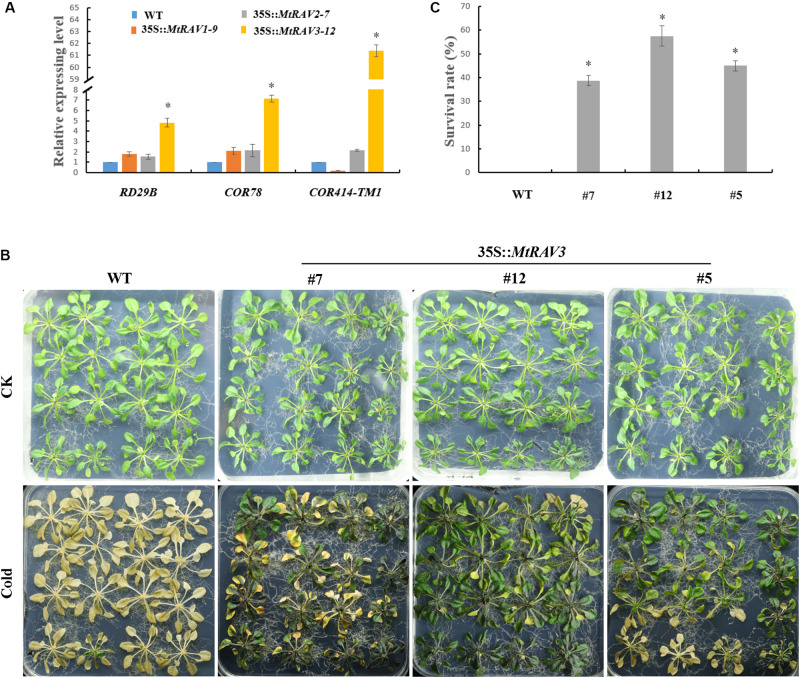
Phenotypic comparison of wild type and 35S:*MtRAV* transgenic *Arabidopsis thaliana* under cold treatment. **(A)** Expression analysis of cold-related marker genes in 35S:*MtRAVs* transgenic plants by qRT-PCR: *RD29B*, *COR78*, and *COR414-TM1*. **(B)** Wild-type and 35S:*MtRAV3* transgenic *Arabidopsis thaliana* were grown on 1/2 MS medium containing 1% agar and grown at 22°C for an additional 18-day. The plates were transferred to a -15°C growth chamber and incubated for 2 h, and then returned to normal growth for 24 h at 22°C. **(C)** After 24 h normal growth after the -15°C cold treatment, we counted the rate of surviving seedlings. This rate was expressed as a percentage (%). Independent *t*-tests demonstrated that there was significant difference (^∗^*P* < 0.05).

According to expression pattern analysis of the three *MtRAV* genes, *MtRAV3* was more sensitive to a low temperature of 4°C, with a rapid increase in expression level. Some transcription factors, such as MYB73, RAV1, CRF2, MYB44, ERF6, WRKY33, and CRF3, are considered to be CBF-independent, first-wave transcription factors that act after cold treatment, playing important roles in cold acclimation (Park et al., 2015). We next investigated the induced expression level of cold-related genes in 35S:*MtRAV* transgenic *Arabidopsis thaliana* plants (Figure 5). We selected three genes: *RD29B* (Responsive to desiccation), *COR78* (cold-regulated), and *COR414-TM1* and analyzed expression of these genes in the *MtRAV1*, *MtRAV2*, and *MtRAV3* transgenic plants by qRT-PCR (Figure 5A and Supplementary Figure S2). All quantitative analysis were performed on three independent biological replicates. The *RD29B* expression levels was markedly up-regulated nearly fivefold in *MtRAV3*-12 transgenic plants, compared to that in WT, and the level was also significantly higher than the expression in *MtRAV1*-9 and *MtRAV2*-7. The expression levels of *COR78* and *COR414-TM1* were also significantly increased, sevenfold and 61-fold in *MtRAV3* transgenic plants, respectively. In contrast, the expression of *COR414-TM1* was down-regulated in *MtRAV1* transgenic plants, compared to that in WT. We further examined the tolerance of three *MtRAV*-overexpresssion transgenic *Arabidopsis thaliana* plants to cold treatment. Only the *MtRAV3* transgenic plants showed strong cold tolerance ability (Figure 5B), while the *MtRAV1* or *MtRAV2* transgenic plants did not show significant difference (data not shown) compared with those of the wild type. After cold acclimation, we observed that wild-type Col showed greater freezing sensitivity compared to the lines of *MtRAV3*. The Col appeared withered and yellow, consistent with freezing that is leading to death. The *MtRAV3* showed strikingly stronger tolerance than WT to freezing stress. The survival rates of *MtRAV3* transgenic plants were significantly higher than that of WT (Figure 5B). These results clearly showed that overexpression of *MtRAV3* enhanced the cold tolerance of transgenic *Arabidopsis thaliana* and the effects on the expression of stress-responsive genes *RD29B*, *COR78*, and *COR414-TM1* implied the importance of RAV protein in cold acclimation.

### Overexpression of *MtRAVs* Improved *Arabidopsis thaliana* Tolerance to Osmotic Stress and High Salinity

Since *MtRAV* transgenic seedlings showed different tolerance to cold and only *MtRAV3* transgenic seedlings showed significant cold resistance, we next explored the phenotypes of *MtRAV* transgenic *Arabidopsis thaliana* under osmotic stress and salt stress.

**FIGURE 6:**
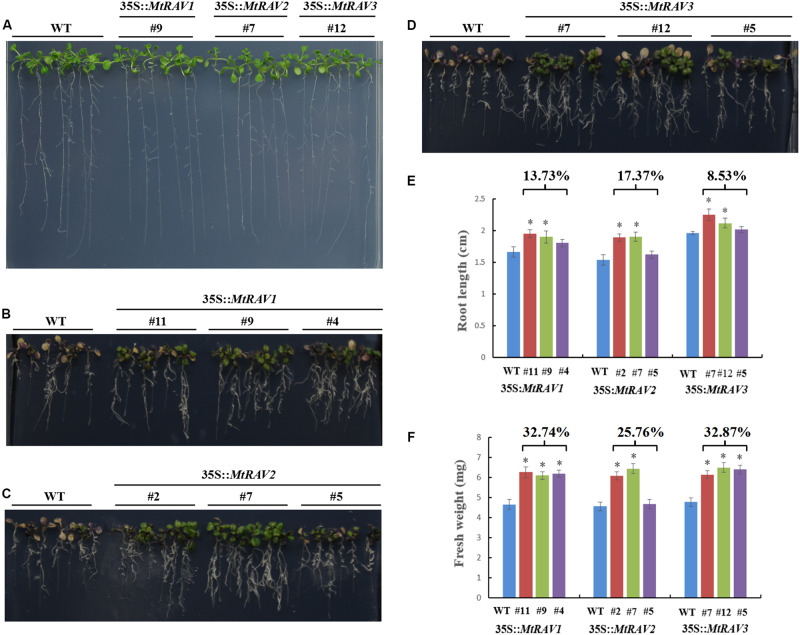
Phenotypic comparison of wild type and 35S:*MtRAVs* transgenic plants under 500 mM mannitol treatment. **(A)** 5-day-old seedlings of three transgenic lines and WT *Arabidopsis thaliana* were planted on 1/2 MS medium without mannitol for 7 days. **(B–D)** 5-day-old seedlings of three transgenic lines and WT *Arabidopsis thaliana* were planted on 1/2 MS medium with 500 mM mannitol for 7 days. **(E)** The root lengths of *MtRAVs* transgenic lines and WT were measured 7 days after 500 mM mannitol treatment. The percentage represents the average increase of root length in *MtRAV* transgenic seedlings compared to WT, for 500 mM mannitol treatment. **(F)** The fresh weight of *MtRAV* transgenic lines and WT were measured 7 days after 500 mM mannitol treatment. The percentage represents the average increase of fresh weight in *MtRAV* transgenic seedlings compared to WT, under 500 mM mannitol treatment. Independent *t*-tests demonstrated that there was significant difference (^∗^*P* < 0.05).

All lines showed similar phenotypes in response to osmotic stress when grown on 1/2 MS medium (Figure 6A). However, with exposure to 500 mM mannitol for 7 days (Figure 6), the WT seedlings gradually became yellow and purple. The root length was also shorter than that of transgenic lines (Figures 6B–D). The root lengths of the *MtRAV1*, *MtRAV2*, and *MtRAV3* transgenic plants were 13.73, 17.37, and 8.53% longer than that of wild-type (Figure 6E), respectively. Meanwhile, there was significant increase of fresh weight in the *MtRAV1*, *MtRAV2*, and *MtRAV3* transgenic plants, 32.74, 25.76, and 32.87% compared to wild-type plants (Figure 6F). Collectively, *MtRAVs* transgenic plants were more tolerant than wild-type plants to osmotic stress caused by mannitol at the seedling stage.

**FIGURE 7:**
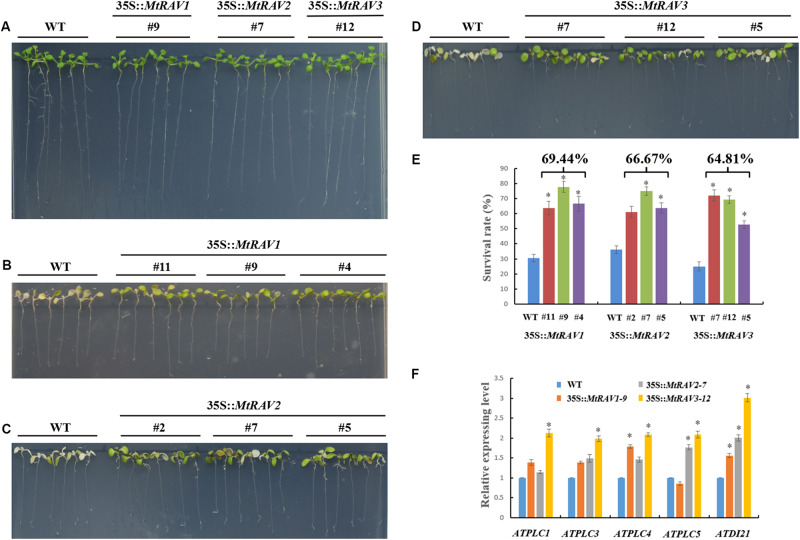
Responses of wild type and 35S:*MtRAVs* transgenic plants to 200 mM NaCl treatment. **(A)** 5-day-old seedlings of three transgenic lines and WT *Arabidopsis thaliana* were planted on 1/2 MS medium without NaCl for 3 days. **(B–D)** 5-day-old seedlings of three transgenic lines and WT *Arabidopsis thaliana* were planted on 1/2 MS medium with 200 mM NaCl for 3 days. **(E)** The survival rate of *MtRAVs* transgenic lines and WT were measured 3 days after 200 mM NaCl treatment. The percentage represents the average survival rate in *MtRAVs* transgenic seedlings under 200 mM NaCl treatment. **(F)** Expression analysis of adversity related marker genes in 35S:*MtRAVs* transgenic plants compared to WT; levels of *ATDI21*, *ATPLC1*, *ATPLC3*, *ATPLC4*, and *ATPLC5* were determined. Independent *t*-tests demonstrated that there was significant difference (^∗^*P* < 0.05).

In the salt treatment assay, 5-day-old seedlings were placed on 1/2 MS medium containing 200 mM NaCl for 3 days. The leaves of wild-type plants gradually became bleached or died, but the vast majority of leaves of 35S:*MtRAVs* transgenic *Arabidopsis thaliana* remained green, indicating they were less affected by NaCl. In the seedling stage, the survival rates were measured as 69.4, 66.7, and 64.8%,for the *MtRAV1*, *MtRAV2*, and *MtRAV3* transgenic plants compared to wild-type plants as the control (Figure 7E). Collectively, the results suggested that MtRAV proteins may participate in plant response to high salinity, and overexpression of *MtRAV* genes contributes to the enhanced tolerance to salt stress during the seedling stage.

To further explore the regulation mechanism of *MtRAVs* on osmotic stress and salt stress, we next examined the expression of some marker genes related to abiotic stress by qRT-PCR, including *ATDI21*, *AtPLC1*, *AtPLC3*, *AtPLC4*, and *AtPLC5* (Figure 7 and Supplementary Figure S2), induced by abiotic stress. All quantitative analysis were performed on three independent biological replicates. *AtPLC3* and *AtPLC4* were measured in *MtRAV1*, *MtRAV2*, and *MtRAV3* transgenic plants and compared to WT. The expression of the *AtPLC1* gene was almost unchanged in *MtRAV2* transgenic plants, and the expression of *AtPLC5* was slightly down-regulated in *MtRAV1* transgenic plants. However, the expression levels of *AtPLC1*, *AtPLC3*, *AtPLC4* and *AtPLC5* were all increased in *MtRAV3* transgenic *Arabidopsis thaliana* plants compared to wild type. The previous results revealed that the up-regulated expression of *ATDI21* was conducive to drought tolerance in *Arabidopsis thaliana* (Fang et al., 2015). There was increased expression of *AtDI21* in *MtRAV1*, *MtRAV2*, and *MtRAV3* transgenic plants. There was a greater than twofold effect for *AtDI21* in *MtRAV3* transgenic plants, with a comparatively lower effect (<2-fold) in *MtRAV1* transgenic plants. The results indicate that the overexpression of *MtRAV* genes induced the expression of *AtPLC1*, *AtPLC3*, *AtPLC4*, *AtPLC5*, and *ATDI21* in *Arabidopsis thaliana*, with the largest effect observed for the *MtRAV3* transgenic plants.

### Overexpression of *MtRAVs* Resulted in the Transgenic *Arabidopsis thaliana* Hyposensitive to Exogenous ABA

**FIGURE 8:**
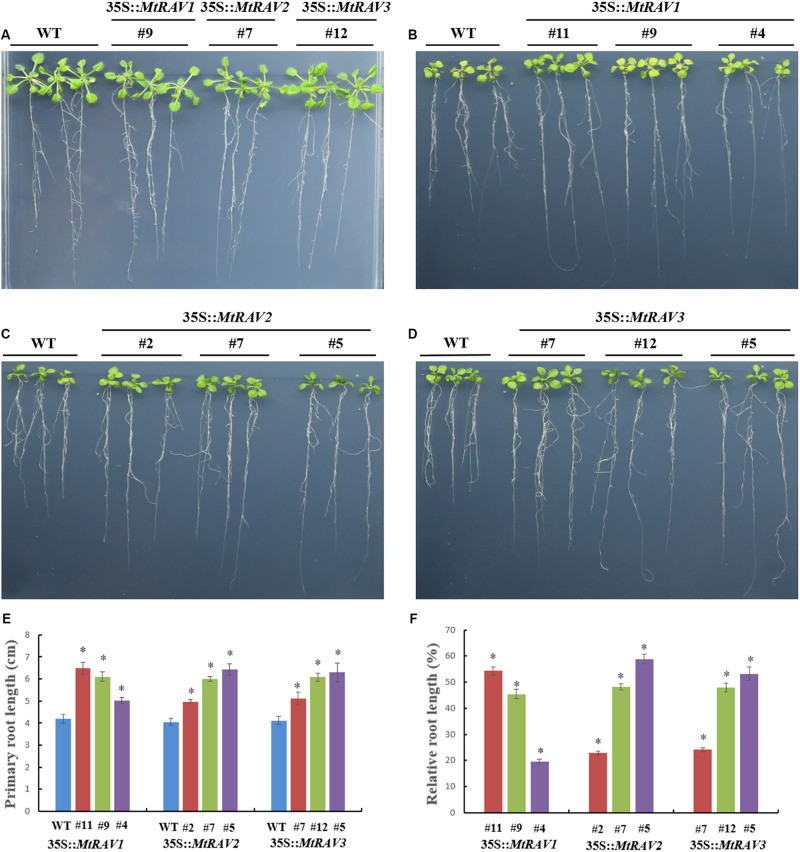
Phenotype assay of 35S:*MtRAVs* transgenic *Arabidopsis thaliana* seedlings grown in MS medium, containing ABA. **(A)** 5-day-old seedlings of the three transgenic lines and WT *Arabidopsis thaliana* were planted on 1/2 MS medium without ABA for 12 days. **(B–D)** 5-day-old seedlings of three transgenic lines and WT *Arabidopsis thaliana* were planted on 1/2 MS medium with 20 μm ABA for 12 days. **(E)** The primary root length of *MtRAVs* transgenic lines and WT were measured 12 days after 20 μm ABA treatment. **(F)** The relative root lengths show an increase of primary root length in *MtRAVs* transgenic seedlings compared to WT. Independent *t*-tests demonstrated that there was significant difference (^∗^*P* < 0.05).

Because exogenous ABA increased MtRAV gene expression and ABA is involved in regulation of stress response pathways, we next investigated whether overexpression of *MtRAV* genes affects the response of plants to exogenous ABA. We examined response of plant growth to ABA for three homozygous *MtRAVs* transgenic lines. When germinated on 1/2 MS medium, all lines showed similar growth. However, in the presence of exogenous ABA (MS + ABA), the growth of both WT and *MtRAV* transgenic seedlings were distinctly inhibited, but the degree of inhibition was greater for the WT than for the *MtRAVs* transgenic plants (Figure 8). The root lengths of *MtRAV* transgenic seedlings were significantly longer than those of WT. The transgenic seedlings of the three *MtRAVs* all displayed an ABA-hyposensitive phenotype. These results suggest that *MtRAV* genes may be involved in the regulation of abiotic stress in an ABA-independent manner.

### Overexpression of *MtRAVs* Increase the Branch Number of *Arabidopsis thaliana*

**FIGURE 9:**
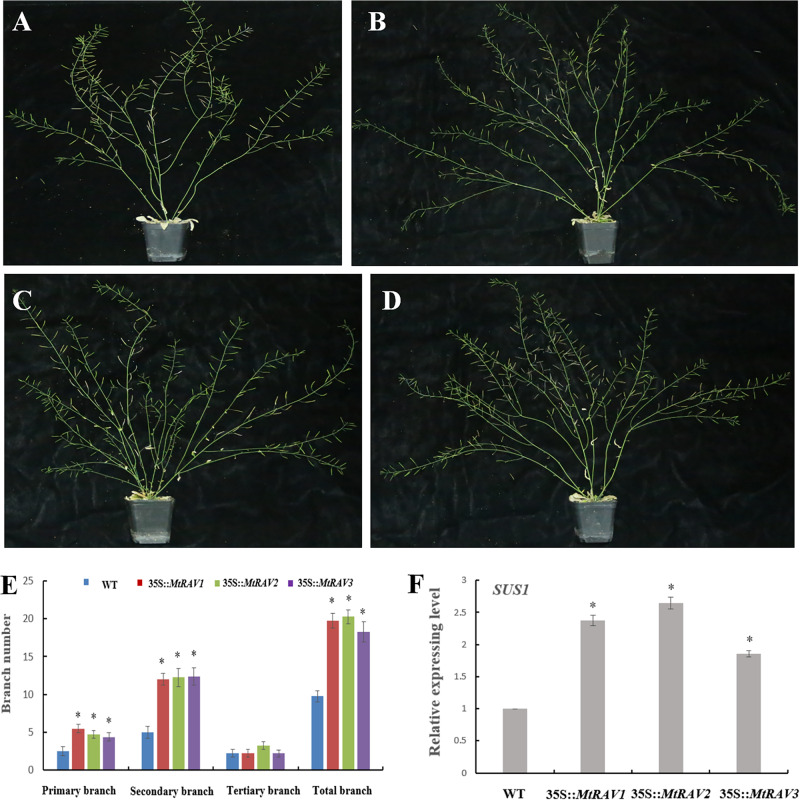
Phenotype of *MtRAVs* transgenic plants and statistical analysis of branch number compared to wild type *Arabidopsis thaliana*. **(A)** Wild type *Arabidopsis thaliana* grown under normal conditions. **(B)** 35S:*MtRAV1-9* transgenic *Arabidopsis thaliana* grown under normal conditions. **(C)** 35S:*MtRAV2-7* transgenic *Arabidopsis thaliana* grown under normal conditions. **(D)** 35S:*MtRAV3-12* transgenic *Arabidopsis thaliana* grown under normal conditions. **(E)** The number of branches for WT and 35S:*MtRAVs* transgenic plants. **(F)** The expression level of *AtSUS1* in *MtRAVs* transgenic *Arabidopsis thaliana*. Independent *t*-tests demonstrated significant difference (^∗^*P* < 0.05).

In addition to the regulatory abiotic stress described above, *MtRAV* transgenic *Arabidopsis thaliana* also exhibit a growth phenotype characterized by increased branch number. Some *MtRAV* transgenic lines of higher expression level showed increased bolting compared with wild type (Figure 9 and Supplementary Figure S3). We analyzed the statistical results of primary branch, secondary branch, and tertiary branch for these lines (*MtRAV1*-9, *MtRAV2*-7 and *MtRAV3*-12, respectively). The number of primary branches compared to WT exhibited a 0.76∼1.2-fold increase and secondary branches increased 1.4∼1.48-fold in *MtRAVs* transgenic lines for plants grown in normal conditions. There was not a significant difference in the number of tertiary branch of transgenic plants compared to that of WT. The total number of branches showed a significantly increase in *MtRAV1*, *MtRAV2*, and *MtRAV3* overexpression lines compared to controls, in a range of 0.87∼1.07-fold. We next analyzed the expression pattern of the *AtSUS1* gene in plants with *MtRAV* overexpression (Figure 9 and Supplementary Figure S2). All quantitative analysis were performed on three independent biological replicates. As previously shown, the *SUS* gene may be related with shoot branching (Poovaiah et al., 2015). In normal growth conditions, the expression of *AtSUS1* in line *MtRAV2*-7 was obviously up-regulated (2.64-fold), with 2.3- and 1.8-fold increase of *AtSUS1* gene expression in lines *MtRAV1*-9 and *MtRAV3*-12, respectively. These results suggest that *MtRAV1, MtRAV2*, and *MtRAV3* positively regulate the expression of *AtSUS1* gene. MtRAV proteins may be involved in the regulation of shoot architecture process.

## Discussion

RAV transcription factors belonging to the AP2/EREBP or B3 superfamily, which exists only in plants. Previous studies were mostly functional explorations of individual RAV genes in different species. Herein, we identified three *MtRAV* genes in *M. truncatula*, and named these three genes *MtRAV1*, *MtRAV2*, and *MtRAV3*. *MtRAV1*, *MtRAV2*, and *MtRAV3* genes encode proteins of 384, 378, and 298 amino acids (Supplementary Figure S1), respectively. There is 86.1% homology between MtRAV1 and MtRAV2, and MtRAV3 has only 45.5 and 46.5% homology with MtRAV1 and MtRAV2 (Figure 1B), respectively. The MtRAV3 protein is smaller in length than MtRAV1 and MtRAV2, and MtRAV3 also exhibits lower homology, compared to MtRAV1 and MtRAV2. The expression level of *MtRAV3* was lower than the expression levels of *MtRAV1* and *MtRAV2* in *M. truncatula* plants grown in normal conditions. There may be functional differences in the *MtRAV* genes, and *MtRAV3* may differ from the others. Similarly, a difference was reported for *Glycine max* GmRAV genes (Zhao et al., 2017).

Previous studies indicated that *Glycine max* RAVs, *Capsicum annuum* RAV1, *Gossypium hirsutum* RAV1, *Zea mays* RAV1, and *Arabidopsis thaliana* RAV1 proteins are localized in the cell nucleus (Lee et al., 2010; Woo et al., 2010; Min et al., 2014; Li et al., 2015; Zhao et al., 2017). Green fluorescence of the three pSAT6:*MtRAVs*-GFP fusion proteins were observed in the cell nuclei, consistent with the localization result of RAV transcription factors in other plants.

The cis-element sequences of a gene promoter are related to the regulatory functions of the gene in plant growth. Our analysis revealed different cis-elements in the promoters of the three *MtRAV* genes, although they all contained AP2 and B3 domains. This suggested that MtRAV1, MtRAV2, and MtRAV3 proteins may have different functions in plant growth and development, even in the regulation of the stress signaling pathway. The promoters of *MtRAV* genes mainly contained MYC, MYB, ABRE, TC-rich repeats, BOX4, and CGTCA-motif elements. The MYC element is involved in signaling pathways related to cold regulation (Chinnusamy et al., 2003). The MYB element is involved in the responses of drought and salt stress (Dubos et al., 2010). And ABRE element is involved in plant responses to drought and ABA through their interaction with ABRE binding proteins (Sakuraba et al., 2014; Hwang et al., 2019). BOX4 elements are photosynthetic response elements, TC-rich repeats elements are involved in defense and stress responsiveness, and CGTCA-motifs are related to MeJA-responsiveness (Ciolkowski et al., 2008). Overall, some transcription factors may affect expression of MtRAVs to regulate plant growth, development, and interaction with the environment.

According to the cis-element analysis of *MtRAVs* gene promoters, we further investigate the expression variation of *MtRAVs* by qRT-PCR in the response to various abiotic stress treatments. As predicted, our results revealed increased expression of *MtRAV3* from the start of 4°C treatment, reaching a level 154-fold higher than the un-treated control at 24 h. The amounts of the three *MtRAVs* transcripts were rapidly induced within 1 h of NaCl treatment, and the expression of *MtRAV3* also markedly increased under PEG treatment. The expression profile of *ZmRAV1* in *Zea mays* in response to 4°C cold treatment immediately increased after treatment (Min et al., 2014), however, *CARAV1* to cold exhibited a gradual increased after 6 h of cold (4°C) treatment in *Capsicum annuum* (Lee et al., 2010). *Zea mays ZmRAV1*, *Gossypium hirsutum GhRAV1*, *Capsicum annuum CaRAV1*, and *Brassica napus BnaRAV-1-HY15* were also induced by NaCl, dehydration, and PEG treatments in different stages of growth and development (Lee et al., 2010; Zhuang et al., 2011; Min et al., 2014; Li et al., 2015). However, a separate report showed transcriptional down-regulation of two *Arabidopsis thaliana RAV* genes (*AtRAV1L and AtRAV2*) by drought and salt stress (Fu et al., 2014). Overall, these results suggest that MtRAVs are involved in the regulatory pathways of abiotic stress.

We next examined the tolerance of *MtRAV* transgenic *Arabidopsis thaliana* to abiotic stress. To do this, we first examined the expression level of cold-related marker genes, including *RD29B*, *COR78*, and *COR414-TM1*, which were markedly up-regulated in *MtRAV3* transgenic plants under normal growth conditions compared to the levels in *MtRAV1*, *MtRAV2*, and wild type plants. We next observed that only *MtRAV3* transgenic *Arabidopsis thaliana* plants displayed significant cold to lerance ability, compared to *MtRAV1* and *MtRAV2* transgenic *Arabidopsis thaliana*, as well as WT. Overexpression of *SlRAV2* in *Solanum lycopersicum* resulted in stronger tolerance ability to cold stress (Li et al., 2011). The *ICE*–*CBF*–*COR* signaling pathway is considered important for cold resistance of plants (Liu et al., 2019). CBFs activate downstream *COR* genes (Chinnusamy et al., 2003; Park et al., 2015; Zhao C. et al., 2016; Kidokoro et al., 2017). And other transcription factors which are induced during cold acclimation in *Arabidopsis thaliana*, such as *HSFC1*, *ZAT12*, and *CZF1*, are also regulate the expression of *COR* genes (Zhao C. et al., 2016; Shi et al., 2017). Overexpression of these genes could induce the expression of *COR* genes even in normal growth conditions, implying that they could directly regulate *COR* genes (Park et al., 2015). And *AtCBF1* is involved in the regulation of RAV family and ERF family (Li et al., 2011). Thus, the *MtRAVs* may directly regulate cold-related genes, such as *RD29B*, *COR78*, and *COR414-TM1*. *MtRAV3* may have stronger regulation ability than *MtRAV1* and *MtRAV2* in the cold regulation pathway, but this should be investigated further.

Our results revealed that all three *MtRAVs* transgenic *Arabidopsis thaliana* lines increased resistance to salt and osmotic stress to different degrees. Similarly, overexpression *Arabidopsis thaliana* plants of *CaRAV1*, *GmRAV-03*, and *ZmRAV1* showed good tolerance to high salinity and osmotic stress compared to wild type plants (Lee et al., 2010; Min et al., 2014; Zhao et al., 2017). Additionally, the overexpression of *ZmRAV1* increased the survival rate and primary root length of *Arabidopsis thaliana*, and decreased relative electrolyte leakage under stress condition (Min et al., 2014). Transgenic *Gossypium hirsutum* expressing *AtRAV1/2* exhibited resistance to drought stress under field and greenhouse conditions, by scavenging reactive oxygen species and osmotic adjustment (Mittal et al., 2014). However, *Gossypium hirsutum GhRAV1* transgenic *Arabidopsis thaliana* exhibited greater sensitivity than wild type plants for NaCl and PEG stress (Li et al., 2015). Thus, there may be species-specific differences in the functions of *RAV* genes. We next examined the expression of some marker genes related to abiotic stress by qRT-PCR, including *ATDI21*, *AtPLC1*, *AtPLC3*, *AtPLC4*, and *AtPLC5*, induced by abiotic stresses (Hirayama et al., 1995; Lee et al., 2005; Tasma et al., 2008; Fang et al., 2015). The results showed that *MtRAVs* induced the expression of these genes to different degrees. Increased expression levels of *ATDI21*, *AtPLC1*, *AtPLC3*, *AtPLC4*, and *AtPLC5* were observed in *MtRAV3* transgenic *Arabidopsis thaliana* plants compared to wild type. Overall, the results suggested different regulatory functions of *MtRAVs* in response to different abiotic stresses.

As suggested by the previous cis-element analysis of *MtRAV* promoters, MtRAVs may be involved in ABA signaling pathways. All *MtRAV* transgenic *Arabidopsis thaliana* were hyposensitive to ABA, but the three *MtRAV* genes were affected to different degrees by ABA, compared to control plants without ABA treatment. Previous studies reported that some stress-responsive genes enhance tolerance to stresses through the ABA-signaling pathway, using both ABA-dependent and ABA-independent methods (Dietz et al., 2010; Fu et al., 2014; Sato et al., 2018). For example, the ABA-PYL-PP2Cs-SnRK2s-AtRAV1-NAC-SAGs signal pathway increased tolerance of stress by causing water to preferentially flow to developing tissues (Zhao Y. et al., 2016). Other *RAV* genes also showed increased expression in response to ABA, including *GhRAV1* and *GmRAV-03*, but their transgenic *Arabidopsis thaliana* were insensitive to exogenous ABA (Finkelstein and Lynch, 2000; Li et al., 2015; Zhao et al., 2017). MtRAVs may be involved in ABA signaling pathways through ABA-independent methods.

Previous RAV studies suggested that MtRAVs may act in plant growth and development. The T3 *MtRAV* transgenic *Arabidopsis thaliana* revealed a significant increase in the total number of branches in the normal growth condition compared to WT. However, this phenotype is different for other *RAV* transgenic *Arabidopsis thaliana*, with reduced lateral roots and rosette leaf reported in *AtRAV1*-overexpression plants (Hu et al., 2004), strong growth inhibition in *AtRAV1*-overexpressing transgenic plants (Fu et al., 2014), and late flowering of *TEM* overexpressing transgenic plants (Castillejo and Pelaz, 2008). Interestingly, *Castanea sativa CsRAV1* induced the early formation of sylleptic branches in hybrid poplar *Populus tremula* × *P. alba* plantlets. We found 1.8∼2.6-fold increased expression of the sucrose synthase (*SUS*) gene in *MtRAV* transgenic *Arabidopsis thaliana* plants compared to the level in WT. Sucrose synthase (SUS) converts sucrose and uridine di-phosphate (UDP) into UDP-glucose and fructose. *Panicum virgatum PvSUS1* transgenic switchgrass showed increased plant tiller number by up to 79% compared to control plants (Poovaiah et al., 2015). These results suggests that a subset of *MtRAVs* positively regulate the expression of *AtSUS1* and possibly regulate axillary bud differentiation. This should be a focus of future work.

As shown above, *MtRAVs* are involved in regulation of abiotic stress and plant growth and development. The functions of *MtRAVs* may be diverse, and there may be some gene-specific functions, especially for *MtRAV3*. Further work is required to explore the functions of *MtRAVs.*

## Conclusion

In summary, three *MtRAV* genes were cloned from *M. truncatula* based on transcriptional factor prediction by PlantTFDB and the *M. truncatula* genome sequence. Constructs encoding these genes were transformed into *Arabidopsis thaliana*. Expression analysis showed a much lower expression level of *MtRAV3* in wild-type *M. truncatula* plants growing in normal conditions than that of *MtRAV1* and *MtRAV2*, but the expression level of *MtRAV3* was significantly increased by application of exogenous low temperature, PEG, and NaCl. The results showed that only *MtRAV3*-overexpressed transgenic plants showed strong cold resistance. *MtRAV* transgenic *Arabidopsis thaliana* plants were hyposensitive to ABA, but more tolerant to high salt stress and osmotic stress. Additionally, increased branch number was observed for *MtRAV* transgenic plants. According to expression analysis and the observations of transgenic plants, the MtRAVs from *M. truncatula* are partly redundant, and the function of *MtRAV3* is more comprehensive. This study lays the foundation for application of *MtRAV* genes in legumes.

## Data Availability Statement

All datasets generated for this study are included in the article/Supplementary Material.

## Author Contributions

YS and SW conceived and designed the experiments. SW and TG performed the experiments. SW and RL analyzed the data. QY and JK contributed reagents, materials, and analysis tools. SW wrote the manuscript. ZW revised the manuscript. All authors read and approved the final manuscript.

## Conflict of Interest

The authors declare that the research was conducted in the absence of any commercial or financial relationships that could be construed as a potential conflict of interest.

## Abbreviations

ABA: abscisic acid
COR: cold-regulated
*M. truncatula*: Medicago truncatula;
PEG: polyethylene glycol
PLC: Phospholipase C
qRT-PCR: quantitative real-time polymerase chain reaction
RAV: Related to ABI3/VP1.
