# Supplementary material for: Expression of Three Related to ABI3/VP1 Genes in Medicago truncatula Caused Increased Stress Resistance and Branch Increase in Arabidopsis thaliana
Source: Front Plant Sci. 2020 May 25;11:611. doi: 10.3389/fpls.2020.00611 (PMC7261895; doi:10.3389/fpls.2020.00611)
Supplement: Supplementary file 1 [file Data_Sheet_1.docx]

Supplementary Material

**Table S1.** Primer sequences used for gene cloning, plasmid construction and qRT-PCR.

| **Primer name** | **Primer sequence(5′-3′)** | | |
| --- | --- | --- | --- |
|  | **F** | **R** |  |
| **For gene clone** |  |  | |
| MtRAV1P | TGTGAGGGGTAGGAATGAAAATGAAA | CCTGAAACCAAGACTGAGTAGCAGGT | |
| MtRAV2P | CCCTTCCCTTCCATTCCTTATAGTCC | CAGTTATCATTCTCCTTTAATTCCCTTG | |
| MtRAV3P | GATCGGATTGGGATGAAATTATGAAG | TAGAATTGTGGGATAGCTGTGCCTTG | |
| **For GFP fusion** |  |  | |
| SAT-RAV1 | CACCATTTACGAACGATAGCATGGAATTAGGAGGAAGTTCC | CTAGTCAGATCTACCATCCCTAAGGCTCCAATTATCTTTGGC | |
| SAT-RAV2 | CACCATTTACGAACGATAGCATGGATGGAGGTAGTTGCATAG | CTAGTCAGATCTACCATCCCCAAAGCTCCAATAATCTTAG | |
| SAT-RAV3 | CACCATTTACGAACGATAGCATGCTTTCAAATTGGAGGAT | CTAGTCAGATCTACCATCCCGCTGATACTTACGCCAAAGA | |
| **For overexpression** |  |  | |
| 3301-RAV1 | GAACACGGGGGACTCTTGACATGGAATTAGGAGGAAGTTCC | TTTACCCTCAGATCTACCATTAAGGCTCCAATTATCTTTGGC | |
| 3301-RAV2 | GAACACGGGGGACTCTTGACATGGATGGAGGTAGTTGCATAG | TTTACCCTCAGATCTACCATCAAAGCTCCAATAATCTTAG | |
| 3301-RAV3 | GAACACGGGGGACTCTTGACATGGAGTTGATGCAAGAAGTGA | TTTACCCTCAGATCTACCATTTGTTGTTGGTAATTATGATAT | |
| **For qRT-PCR** |  |  | |
| qMtRAV1  MTR_1g093600 | GGAGTCTCGTAAACTTCCATCG | CTCGTAAATCTGTGCTCCCC | |
| qMtRAV2  MTR_5g053920 | CATTACAGAAAGCCGATTGCG | TCCACACTTTCCCTCCAATG | |
| qMtRAV3  MTR_1g116920 | GATGCTGGCGACATTGTTTC | GGAGTGAAAAGTGTTGCTGATG | |
| qAtSUS1  AT5G20830 | GAGGAGACAGGAGGAAAGAGTCAAAG | AAAGTCGGTAAACCACAAGTCATAGC | |
| qATDI21  AT4G15910 | TGTAACAGCAGCAGGATTGAGTAAGG | CGCACGATTGGAAGGTCTGTAGTATC | |
| qAtPLC1  AT5G58670 | CGGTTAAGCATGAGTGAGCA | ATGAATCCACCCAACGAGAG | |
| qAtPLC3  AT4G38530 | CAAGGACATGGGAAGCAACT | CTTTTGCAAGGGTCGAAGAG | |
| qAtPLC4  AT5G58700 | AACTTGCTCTGCTCCGTGTT | AAGAGTGGAACAGCGCGTAT | |
| qAtPLC5  AT5G58690 | CAAAAGACATGGGAGCCATT | ACCCGAGAAATCGTCCTTCT | |
| qAtRD29B  AT5G52300 | GTGGAGTGAAGGAGACGCAACAAGGG | TAGTCCCAACGGTGGTGCCAAGTGAT | |
| qAtCOR78  AT5G52310 | CAAACAGAGGAACCACCACTCAACAC | TAATCGGAAGACACGACAGGAAACAC | |
| qAtCOR414-TM1  AT1G29395 | TGTGGTCTGTTATGCTGCTCCGATTT | ATATGCTTTCTCCAAGCTCCCTGCTC | |
| qAtActin  AT1G13440 | ACCACTGTCCACTCTATCACTGC | TGAGGGATGGCAACACTTTCCC | |
| qMtActin  MTR_3g095530 | TGGGCTGCCACAGAACATTTGA | GCTGTGGTTGCTTTTTTGGTGTCTC | |


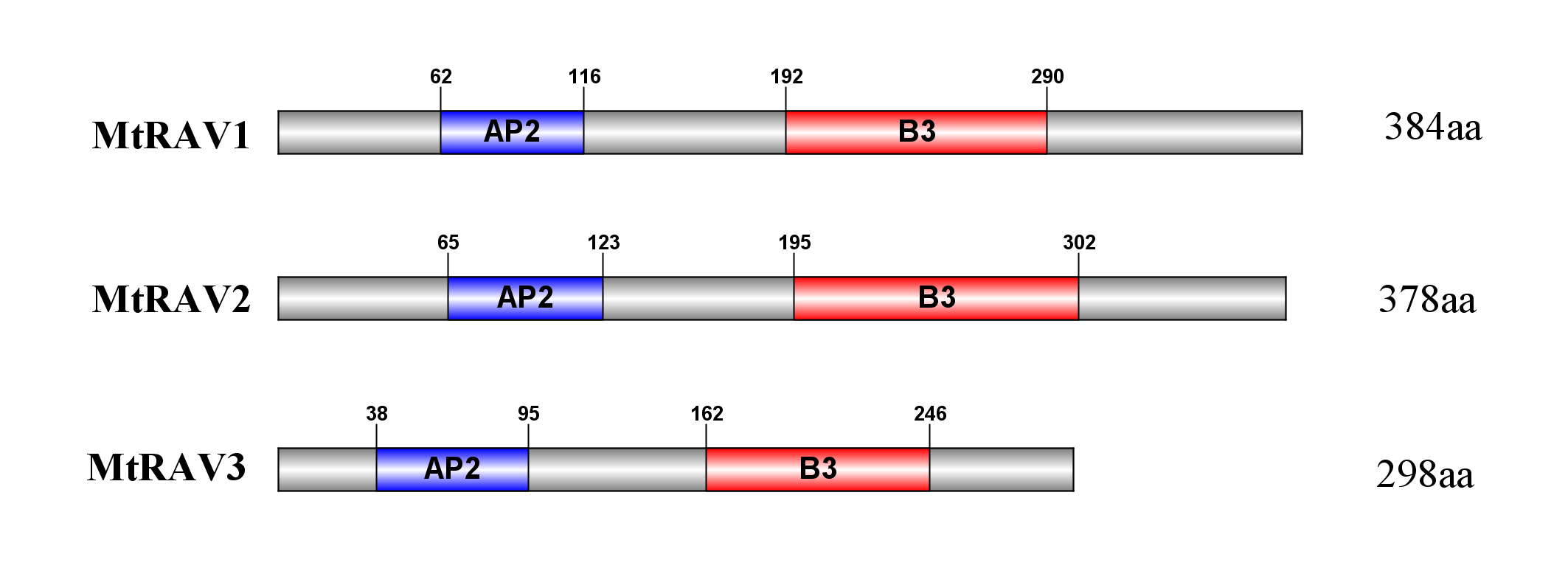


**Figure S1.** Predicted structures of the RAV proteins in *M. truncatula*. The B3 domains and AP2 domains are indicated by the red and blue boxes, respectively.


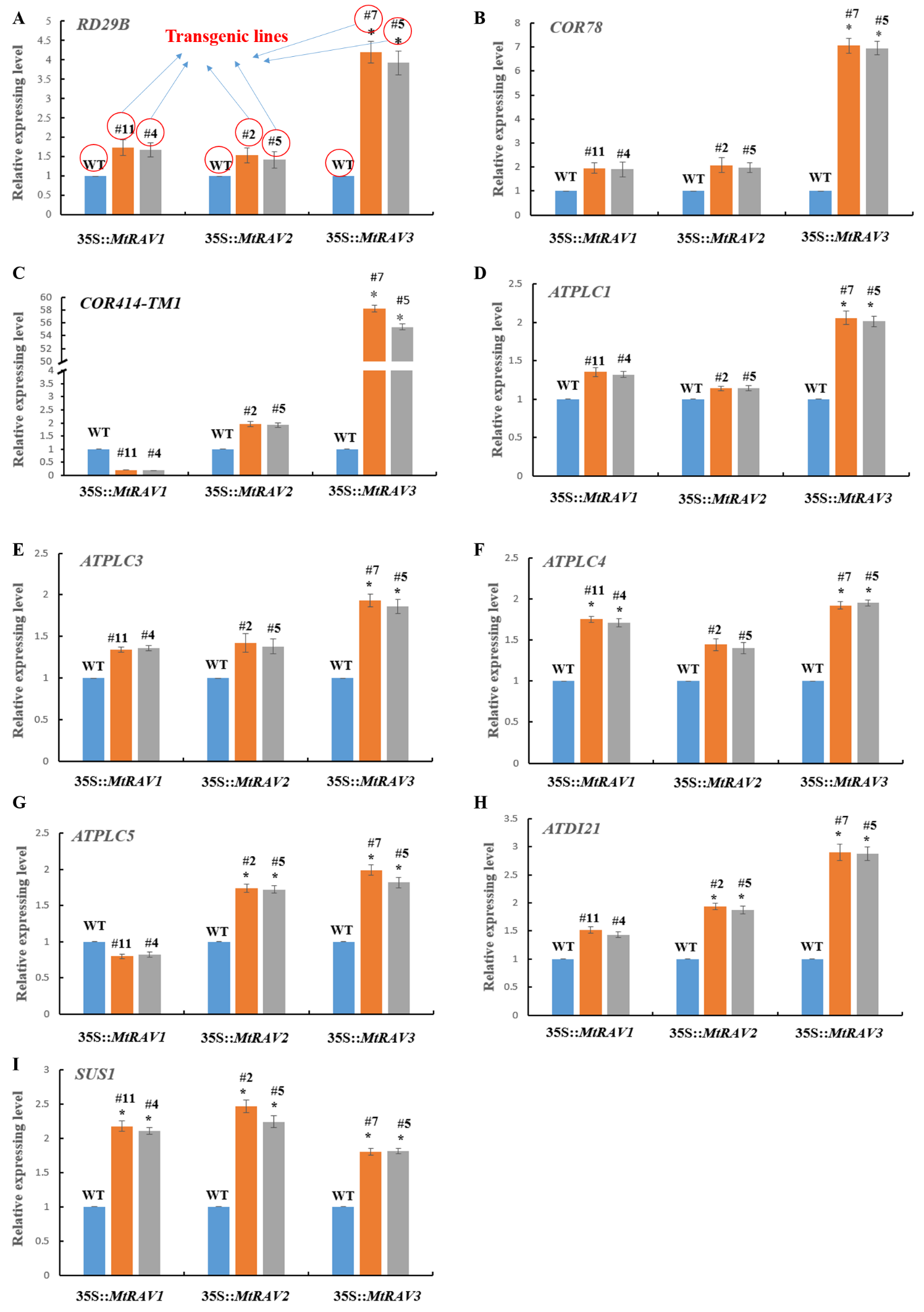


**Figure S2.** The qRT-PCR analysis of some marker genes for different transgenic lines. (A) Expression analysis of cold-related marker gene *RD29B* in 35S::*MtRAVs* transgenic lines compared to WT by qRT-PCR. (B) Expression analysis of cold-related marker gene *COR78* in 35S::*MtRAVs* transgenic lines compared to WT by qRT-PCR. (C) Expression analysis of cold-related marker gene *COR414-TM1* in 35S::*MtRAVs* transgenic lines compared to WT by qRT-PCR. (D) Expression analysis of adversity related marker gene *ATPLC1* in 35S::*MtRAVs* transgenic lines compared to WT by qRT-PCR. (E) Expression analysis of adversity related marker gene *ATPLC3* in 35S::*MtRAVs* transgenic lines compared to WT by qRT-PCR. (F) Expression analysis of adversity related marker gene *ATPLC4* in 35S::*MtRAVs* transgenic lines compared to WT by qRT-PCR. (G) Expression analysis of adversity related marker gene *ATPLC5* in 35S::*MtRAVs* transgenic lines compared to WT by qRT-PCR. (H) Expression analysis of adversity related marker gene *ATDI21* in 35S::*MtRAVs* transgenic lines compared to WT by qRT-PCR. (I)The expression level of *AtSUS1* in *MtRAVs* transgenic lines compared to WT by qRT-PCR. Independent t-tests demonstrated that there was significant difference (∗P < 0.05).


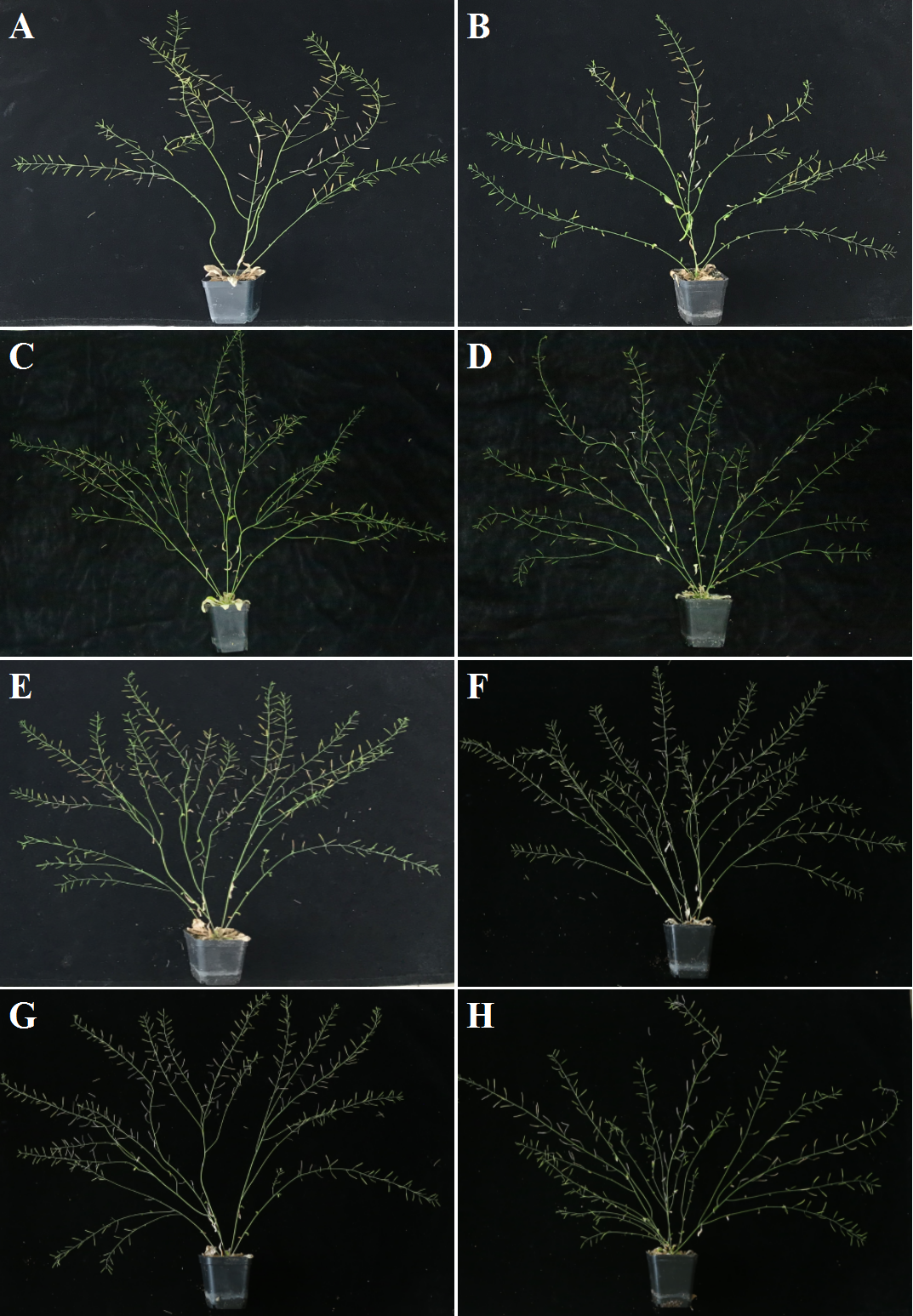


**Figure S3.** The Phenotype of other lines of *MtRAVs* transgenic Arabidopsis. (A and B)Wild type *Arabidopsis* grown under normal conditions. (C) 35S::*MtRAV1-4* transgenic *Arabidopsis* grown under normal conditions. (D) 35S::*MtRAV1-11* transgenic *Arabidopsis* grown under normal conditions. (E) 35S::*MtRAV2-2* transgenic *Arabidopsis* grown under normal conditions. (F) 35S::*MtRAV2-5* transgenic *Arabidopsis* grown under normal conditions. (G) 35S::*MtRAV3-5* transgenic *Arabidopsis* grown under normal conditions. (H) 35S::*MtRAV3-7* transgenic *Arabidopsis* grown under normal conditions.

*
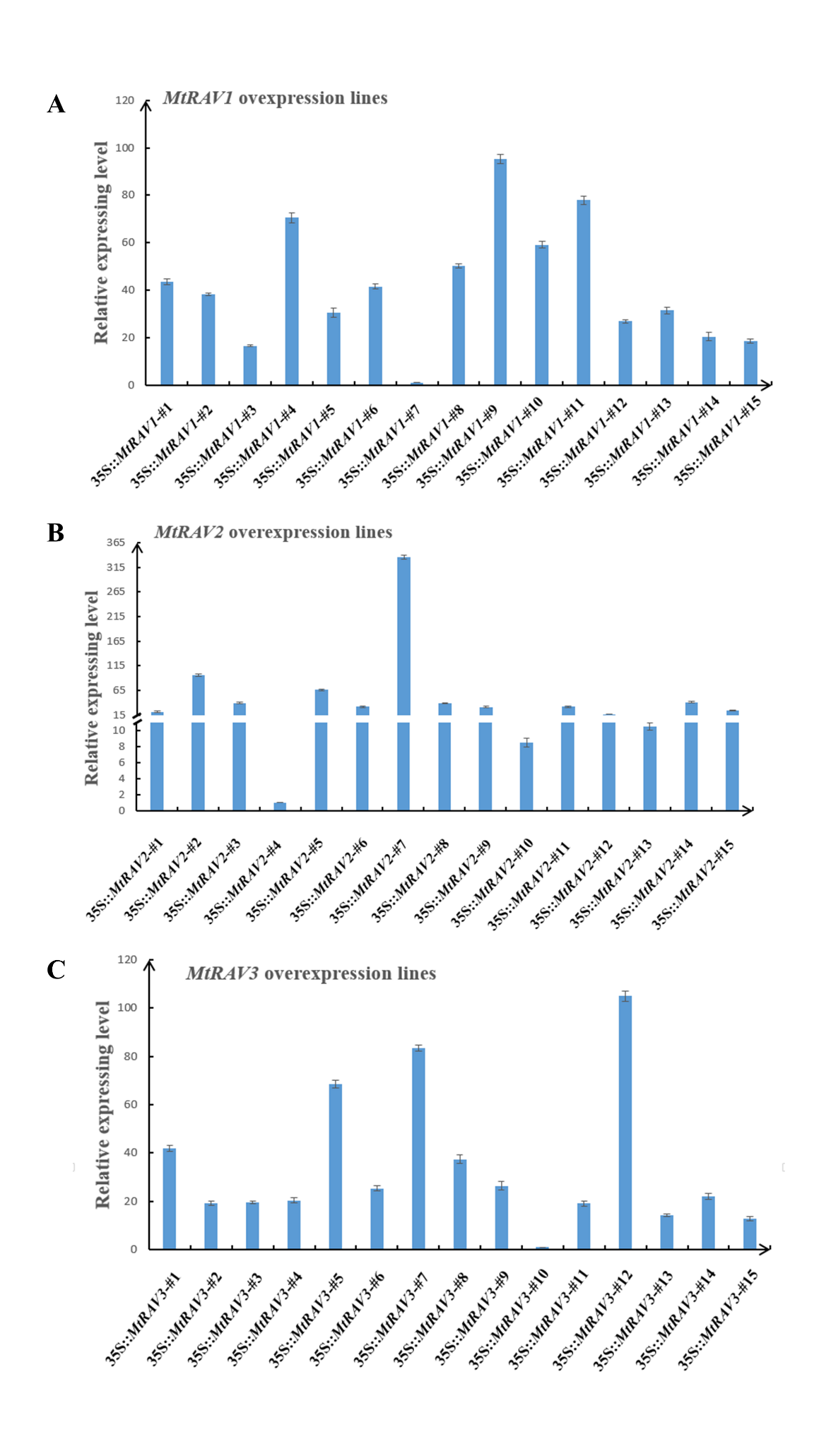
*

**Figure S4. The different transgenic lines of three *MtRAV* genes overexpression in *Arabidopsis thaliana*. (A)The relative expressing level of fifteen *MtRAV1* overexpression transgenic *Arabidopsis thaliana* lines, relative to the lowest expression level of 35S::*MtRAV1*-#7. (B) The relative expressing level of fifteen *MtRAV2* overexpression transgenic *Arabidopsis thaliana* lines, relative to the lowest expression level of 35S::*MtRAV2*-#4. (C) The relative expressing level of fifteen *MtRAV3* overexpression transgenic *Arabidopsis thaliana* lines, relative to the lowest expression level of 35S::*MtRAV3*-#10.**
